# Wogonin Mitigates Depression by Inhibiting TNF‐α/TNFR1/CXCL1 Signalling‐Mediated Astrocyte Activation

**DOI:** 10.1111/jcmm.71116

**Published:** 2026-03-25

**Authors:** Huihui Chai, Kunying Zhao, Weiyan Hu, Bin Liu

**Affiliations:** ^1^ The National Key Clinic Specialty, the Engineering Technology Research Center of Education Ministry of China, Guangdong Provincial Key Laboratory on Brain Function Repair and Regeneration, Department of Neurosurgery, Zhujiang Hospital Southern Medical University Guangzhou China; ^2^ School of Pharmaceutical Science & Yunnan Key Laboratory of Pharmacology for Natural Products Kunming Medical University Kunming China; ^3^ College of Modern Biomedical Industry Kunming Medical University Kunming China; ^4^ Department of Neurosurgery The Tenth Affiliated Hospital of Southern Medical University (Dongguan People's Hospital) Dongguan China

**Keywords:** astrocyte activation, depression, TNF‐α/TNFR1/CXCL1 signalling, wogonin

## Abstract

Wogonin exhibits notable anti‐inflammatory and neuroprotective effects. Current study aimed to investigate the antidepressant potential of wogonin and its underlying mechanisms through in vitro and in vivo experiments. In vitro, wogonin attenuated lipopolysaccharide (LPS)‐induced astrocyte activation. Furthermore, wogonin inhibited the TNF‐α/TNFR1/CXCL1 signalling axis, as evidenced by decreased protein levels of TNF‐α and CXCL1 and reduced interaction between TNF‐α and its receptor TNFR1. The addition of recombinant TNF‐α (r‐TNF‐α) blocked these inhibitory effects of wogonin. In vivo, in a chronic unpredictable mild stress (CUMS) mouse model, wogonin alleviated depression‐like behaviours. Moreover, wogonin rescued CUMS‐induced impairments in hippocampal synaptic plasticity, restoring post‐tetanic potentiation (PTP), long‐term potentiation (LTP) and mitigating neuronal damage in the CA1 region. Further analysis revealed that wogonin reduced levels of TNF‐α and CXCL1 in both the hippocampal CA1 region and cerebrospinal fluid, and decreased the expression of astrocyte activation markers GFAP and C3. These effects of wogonin in vivo were reversed by co‐administration of r‐TNF‐α and were replicated by treatment with the TNF‐α inhibitor etanercept. These results demonstrate that wogonin may exert antidepressant effects by modulating the TNF‐α/TNFR1/CXCL1 pathway, thereby improving neuroinflammation and astrocyte activation.

## 1 Introduction

Depression is primarily characterized by enduring and pronounced feelings of low mood, diminished interest, decreased energy and impaired cognitive functioning [1, 2]. Patients also exhibit a high relapse rate. In China, an estimated 95 million people struggle with depression, which is increasingly affecting younger generations. As the pace of modern society accelerates and social competition intensifies, the incidence of depression is expected to increase. Despite the availability of various antidepressant treatments, a significant proportion of patients exhibit limited response or bear the burden of side effects, highlighting the urgent need for novel therapeutic agents.

Growing evidence suggests that neuroinflammation is a key contributor to the pathogenesis of depression [3, 4]. Peripheral inflammatory biomarkers are elevated in patients with depression, with their levels positively correlated with the severity of specific symptoms [5]. Neuroimaging studies have demonstrated the presence of neuroinflammation across multiple brain regions in patients with depression [3, 6]. These clinical findings are further corroborated by animal models, which recapitulate both neuroinflammatory changes and depression‐like behaviours [7]. Importantly, both pharmacological and neuromodulation interventions have been shown to alleviate depressive symptoms while ameliorating inflammation [3, 8]. These evidences shift the focus towards anti‐inflammatory strategies. Therefore, natural products with anti‐neuroinflammatory properties, such as the wogonin derived from *Scutellaria baicalensis*, have emerged as promising candidates for intervention.

Wogonin possesses a broad spectrum of pharmacological properties, notably potent anti‐inflammatory and neuroprotective effects [9]. Its role in mitigating neuroinflammation has been demonstrated across various neurological models. For instance, wogonin was shown to suppress neuro‐inflammation and protect retinal ganglion cells by broadly inhibiting the TLR4‐NF‐κB pathway after optic nerve injury [10]. Furthermore, Wogonin mitigates γ‐irradiation‐induced neurotoxicity in rats by exerting potent antioxidant and anti‐inflammatory effects, significantly reducing oxidative stress and pro‐inflammatory cytokine production while enhancing the Nrf2/HO‐1 defence pathway [11]. However, the mechanism underlying the potential antidepressant effect of wogonin is not fully understood.

Neuroinflammation is often driven by the activation of glial cells, particularly astrocytes [12]. Following central nervous system (CNS) injury, astrocytes undergo activation and changes in their cellular characteristics, transforming into “reactive astrocytes” [13]. This reactive transformation is primarily manifested in three key aspects: Morphologically, the cells undergo hypertrophy, with an enlarged cell body and the extension of more numerous and thicker processes, accompanied by a significant upregulation of the classical marker glial fibrillary acidic protein (GFAP). At the molecular level, reactive astrocytes markedly synthesize and release pro‐inflammatory cytokines, chemokines, along with other neuroinflammatory mediators. Among the complex network of neuroinflammatory mediators, the tumour necrosis factor‐alpha (TNF‐α) signalling pathway has been implicated as a central driver [14, 15]. TNF‐α levels are elevated in depression and correlate with clinical symptoms [16]. In addition, TNF‐α can directly induce synaptic dysfunction and depression‐like behaviours in animal models [17]. A key downstream effector of TNF‐α in astrocytes is the chemokine C‐X‐C motif ligand 1 (CXCL1). CXCL1 induces and recruits neutrophils to the CNS, and participates in neuroinflammatory responses by binding to the main receptor, CXCR2 [18]. CXCL1 is predominantly secreted by activated astrocytes in brain tissue [19]. CXCL1 plays an essential role in regulating hippocampal neuronal plasticity and apoptosis in stress‐induced depression [20]. Therefore, inhibiting the activation of TNF‐α/TNFR1/CXCL1 axis in astrocytes has emerged as a potential therapeutic strategy.

In the present study, we hypothesize that wogonin may regulate CXCL1 production by targeting the TNF‐α signalling pathway. However, whether wogonin modulates the TNF‐α/TNFR1‐mediated CXCL1 signalling axis in astrocytes, and whether this pathway is the key mechanism underlying its antidepressant effects, remain to be elucidated. To test this hypothesis, we employed a chronic unpredictable mild stress (CUMS)‐induced mouse model and lipopolysaccharide (LPS)‐treated astrocyte model to assess the antidepressant efficacy of wogonin and its modulation of the TNF‐α/TNFR1/CXCL1 pathway.

## 2 Materials and Methods

### 2.1 Isolation and Culture of Mouse Cortical Astrocytes

Cell isolation was performed as described by Schildge et al. [21]. Isolated cells were identified through measurement the expression of GFAP using immunofluorescence. Astrocytes at passages three and five were used in subsequent experiments.

### 2.2 Cell Models

To investigate the effect of wogonin on LPS‐induced astrocyte activation, mouse cortical astrocytes were initially treated with LPS (100 ng/mL) for 24 h [22], followed by treatment with various concentrations of wogonin (0, 1, 5, 10, 20 μM; Merk, no. PHL89825, purity> 95%). Cells without any treatment were used as a control. To analyse the effects of wogonin on TNF‐α, TNFR1 and CXCL‐1 secretion in LPS‐induced activated astrocytes, mouse cortical astrocytes were categorized into the following groups: control, LPS, LPS + 5 μM wogonin and LPS + 10 μM wogonin. To examine whether wogonin inhibits astrocyte activation through the TNF‐α/TNFR1/CXCL1 signalling axis, mouse cortical astrocytes were categorized into the following groups: LPS group, LPS + TNF‐α antibody (anti‐TNF‐α, 20 μg/mL) [23], LPS + 10 μM wogonin and LPS + 10 μM wogonin + recombinant TNF‐α (r‐TNF‐α, 5 ng/mL) protein [24].

### 2.3 Immunofluorescence

Following the specified treatments, mouse cortical astrocytes cultured on 12 mm glass coverslips were rinsed with PBS, fixed with 4% formaldehyde for 10 min, and permeabilized with 1% Triton X‐100 for 20 min. Cells were then washed with PBS three times before incubation with primary antibodies against C3 and GFAP overnight at 4°C. Next day, mouse cortical astrocytes were incubated with a secondary antibody solution after washing three times with PBS. Following three more washes with PBS, the cell nuclei were stained with 4′,6‐diamidino‐2‐phenylindole in a light‐free environment. After three additional washes with PBS, the cells were mounted with mounting medium, and the fluorescence signals were observed under a fluorescence microscope. The intensities of C3 and GFAP immunofluorescence signals were semi‐quantitatively analysed by measuring the mean optical density using Image‐Pro Plus 6.0 software (Media Cybernetics, Silver Spring, MD, USA). For normalization, all data are expressed relative to the control and presented as % of control.

### 2.4 Enzyme‐Linked Immunosorbent Assay (ELISA)

CXCL1 and TNF‐α levels in the cell culture supernatant and cerebrospinal fluid were assessed using a mouse CXCL1 ELISA kit (Shanghai Westang Biotechnology Co. LTD, China) and a mouse TNF‐α ELISA Kit (EK282/3–24, MUTI SCIENCES, Hangzhou, China), respectively.

### 2.5 Western Blot

The concentration of proteins enriched from co‐immunoprecipitation, or the total proteins of astrocytes or brain tissues were measured using a BCA Protein Assay kit (Thermo Scientific Pierce). Subsequently, western blot was performed to measure TNF‐α, TNFR1 and CXCL1 levels using the same method reported in our previous article [25]. Primary antibodies for CXCL1, TNF‐α and TNFR1 were purchased from Abcam (Cambridge, MA, USA). Finally, the horseradish peroxidase (HRP) signal on secondary antibody was visualized using Immobilon Western Chemilu HRP substrate and detected on an x‐ray film. The integrated optical density (IOD) of each band was measured using Image‐Pro Plus 6.0 software (Media Cybernetics), and the relative expression level of the target protein was calculated by normalizing its IOD value to that of GAPDH and expressed as a ratio.

### 2.6 Co‐Immunoprecipitation

To investigate the binding interaction between TNF‐α and TNFR1 Co‐immunoprecipitation was conducted to capture both TNF‐α and its associated binding protein using a TNF‐α antibody using the Pierce Co‐immunoprecipitation Kit (Cat. No. 26149, Thermo Scientific Pierce, Rockford, IL, USA), following the guidelines provided. The abundance of TNFR1 and TNF‐α among the enriched proteins was assessed by western blot. The IOD of western blot bands for TNFR1 and TNF‐α was measured in both the immunoprecipitated (IP) samples and the corresponding input lysates. To normalize for differences in protein expression and IP efficiency, the relative TNF‐α/TNFR1 interaction was calculated as follows: relative interaction = (IP‐TNFR1 / IP‐TNF‐α) / (input‐TNFR1 / input‐TNF‐α).

### 2.7 Animal Model

Male C57BL/6 (6–8 weeks) were purchased from GemPharmatech (Foshan, China). All animal experimental procedures were approved by the Experimental Animal Ethics Committee of Guangzhou Laian Technology Co. LTD. A mouse model of depression was induced by exposing mice to CUMS, following the methodology described in our previous studies with minor modifications [20, 25]. Stress factors included fasting (12 h), water deprivation (12 h), cold‐water swimming (5 min), hot‐water swimming (42°C, 5 min), exposure to animal vocalizations (0.5 h), restraint using a self‐developed mouse behavioural restrictor (length 20 cm, diameter 7 cm), circadian rhythm reversal, paired housing, wet cage and cage tilting. Stressors were applied daily over a period of 4 weeks, with 2–3 different stimuli selected each day and presented in an unpredictable sequence to avoid animal adaptation.

Twenty‐eight days after CUMS exposure, mice were divided into 4 groups (*n* = 7): CUMS, CUMS+ wogonin, CUMS+ wogonin + recombinant TNF‐α (r‐TNF‐α) and CUMS+ etanercept (an inhibitor of TNF‐α). During the continuous 42‐day period of CUMS induction, alternate‐day intraperitoneal injections of wogonin (40 mg/kg in 200 μL) [26], etanercept (0.42 mg/kg in 200 μL), or r‐TNF‐α (25 μg/kg) were administered from day 28 to day 42. Mice without CUMS stimulation (*n* = 7) were used as the control group.

### 2.8 Depression‐Like Behaviour Tests

On the first, second and third days after the different interventions, depression‐like behaviour tests, including sucrose preference test (SPT), tail suspension test (TST), forced swimming test (FST), open field test (OFT) and Morris water maze test (MWM) were sequentially conducted using the same methodology as in our prior study [20, 25]. In SPT, the percentage of sucrose preference was calculated. In TST, immobility times during 6 min was recorded. In FST, the immobility time during the final 4 min of the 6‐min test session was recorded. In OFT, travelled total distance was tracked using Visu Track video tracking software. In MWM, the time in the target quadrant and the distance before enter target quadrant was recorded.

### 2.9 Hippocampal Slice Preparation

Mice were anaesthetised with isoflurane and subsequently decapitated. Intact brain tissue was rapidly submerged in ice‐cold (4°C) oxygenated artificial cerebrospinal fluid (1.2 mM MgCl_2_, 125 mM NaCl, 10 mM glucose, 26 mM NaHCO_3_, 1.25 mM KH2PO_4_, 3 mM KCl and 2 mM CaCl_2_). The brain tissues were then sectioned into 350 μm slices using a vibratome. The hippocampal slices were placed into a culture chamber with oxygenated artificial cerebrospinal fluid supplied with 95% O2 and 5% CO_2_ for 1 h before use.

### 2.10 Electrophysiological Recording

Brain slices were placed in the recording chamber and fully immersed in continuously perfused oxygenated artificial cerebrospinal fluid (flow rate, 1–2 mL/min) for recording. Using concentric bipolar electrodes (FHC Inc., USA), the Schaffer collateral in the CA1 region was stimulated, while field excitatory postsynaptic potentials (fEPSPs) were recorded from the radiatum layer of the CA1 region using glass microelectrodes filled with 3 M NaCl (with a resistance of 2–4 MΩ). The positions of the stimulating and recording electrodes were adjusted to obtain input/output (I/O) curves with different stimulation intensities. The stimulation intensity was adjusted to 50% of the maximum value to establish baseline. Following a 30‐min stabilization period, high‐frequency theta‐burst stimulation (TBS) was administered to three trains, each containing five single‐pulse stimulations, followed by 10 triggering stimulations. The training interval was 20 s, triggering stimulation interval was 200 ms and single‐pulse stimulation interval was 10 ms. Long‐term potentiation (LTP) and post‐tetanic potentiation (PTP) and were recorded at 1–5 min and 50–60 min post‐stimulation, respectively. The electrophysiological signals were filtered between 0.1–5 kHz, collected using a Multiclamp 700A amplifier (Molecular Devices, USA), and digitized with a 1322 analog‐to‐digital converter (Molecular Devices, USA). Data analysis was performed using Clampfit software (version 12.0; Molecular Devices, USA).

### 2.11 Immunohistochemistry

Brain slices were incubated in a 60°C oven overnight, after which they were dewaxed, hydrated and underwent antigen retrieval with a 0.0 1 M sodium citrate buffer. After rinsing, the slices were treated with 3% hydrogen peroxide. After rinsing, the slices were blocked with 5% bull serum albumin. Diluted antibodies against TNF‐α, complement C3 (C3) and glial fibrillary acidic protein (GFAP) were used to incubate slices overnight at 4°C. The following day, the slices were rinsed and incubated with secondary antibodies, and stained with 3,3′‐Diaminobenzidine. After haematoxylin staining, differentiation with 1% hydrochloric acid alcohol, and rinsing under running water, dehydration was accomplished using a series of alcohol gradients, followed by immersion in xylene for 10 min. Finally, brain slices were mounted with neutral resin and photographed. The expression levels of TNF‐α, C3 and GFAP were semi‐quantitatively analysed by measuring the mean optical density using Image‐Pro Plus 6.0 software (Media Cybernetics). For normalization, all data are expressed relative to the control and presented as % of control.

### 2.12 Nissl Staining

Following dewaxing and hydration, paraffin brain slices were stained with Nissl staining solution (Beyotime, Shanghai, China) for 5 min After rinsing, the slides were then dehydrated, cleared and mounted. Nissl‐positive neurons were quantified in a blinded manner. Briefly, images captured under 20× magnification were imported into ImageJ (version 1.54p, NIH, National Institutes of Health, Bethesda, MD, USA). Using the “Cell Counter” plugin, an investigator manually counted all neurons that exhibited a clearly defined nucleus, visible nucleolus and intense Nissl substance staining. Cells with pyknotic nuclei or fragmented morphology were excluded. The average count was used for statistical analysis.

### 2.13 Statistical Analysis

Statistical analyses were performed using GraphPad Prism version 9.0 (GraphPad Software, San Diego, CA, USA). Data in bar graphs are presented as the mean ± standard deviation. One‐way analysis of variance was employed to compare differences between more than two groups. Statistical significance was set at *p* < 0.05.

## 3 Results

### 3.1 Wogonin Attenuates LPS‐Induced Astrocyte Activation In Vitro

**FIGURE 1:**
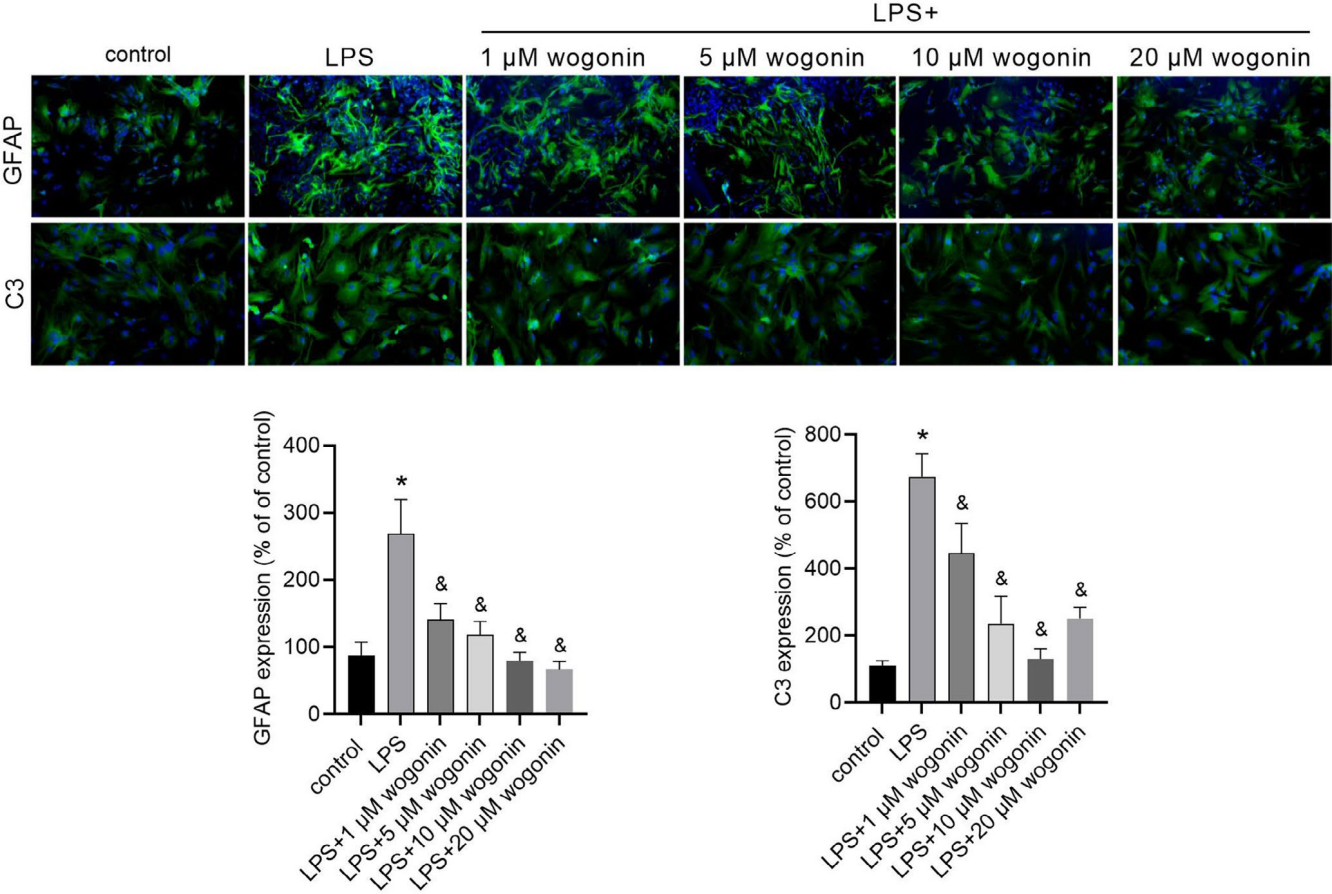
Wogonin at different concentrations suppressed the LPS‐induced upregulation of GFAP and C3. Upper panels show representative images of immunofluorescence and lower panels show statistical results of GFAP and C3 expression level. * indicates *p* < 0.05 compared to the control group. & indicates *p* < 0.05 < LPS group.

Compared to the control group, LPS treatment significantly increased the expression levels of GFAP and C3, indicating successful activation of astrocytes (Figure 1). This effect was markedly attenuated by co‐treatment with wogonin at concentrations of 1, 5, 10 and 20 μM (Figure 1). Notably, the inhibition of C3 expression exhibited a non‐linear trend, with the most pronounced effects observed at 5 μM and 10 μM wogonin, while the effect at 20 μM was slightly attenuated. Based on the overall efficacy and the peak inhibitory effects observed, the concentrations of 5 μM and 10 μM wogonin were selected for subsequent mechanistic experiments.

### 3.2 Wogonin Inhibits LPS‐Induced Activation of the TNF‐α/TNFR1/CXCL1 Signalling Axis In Vitro

**FIGURE 2:**
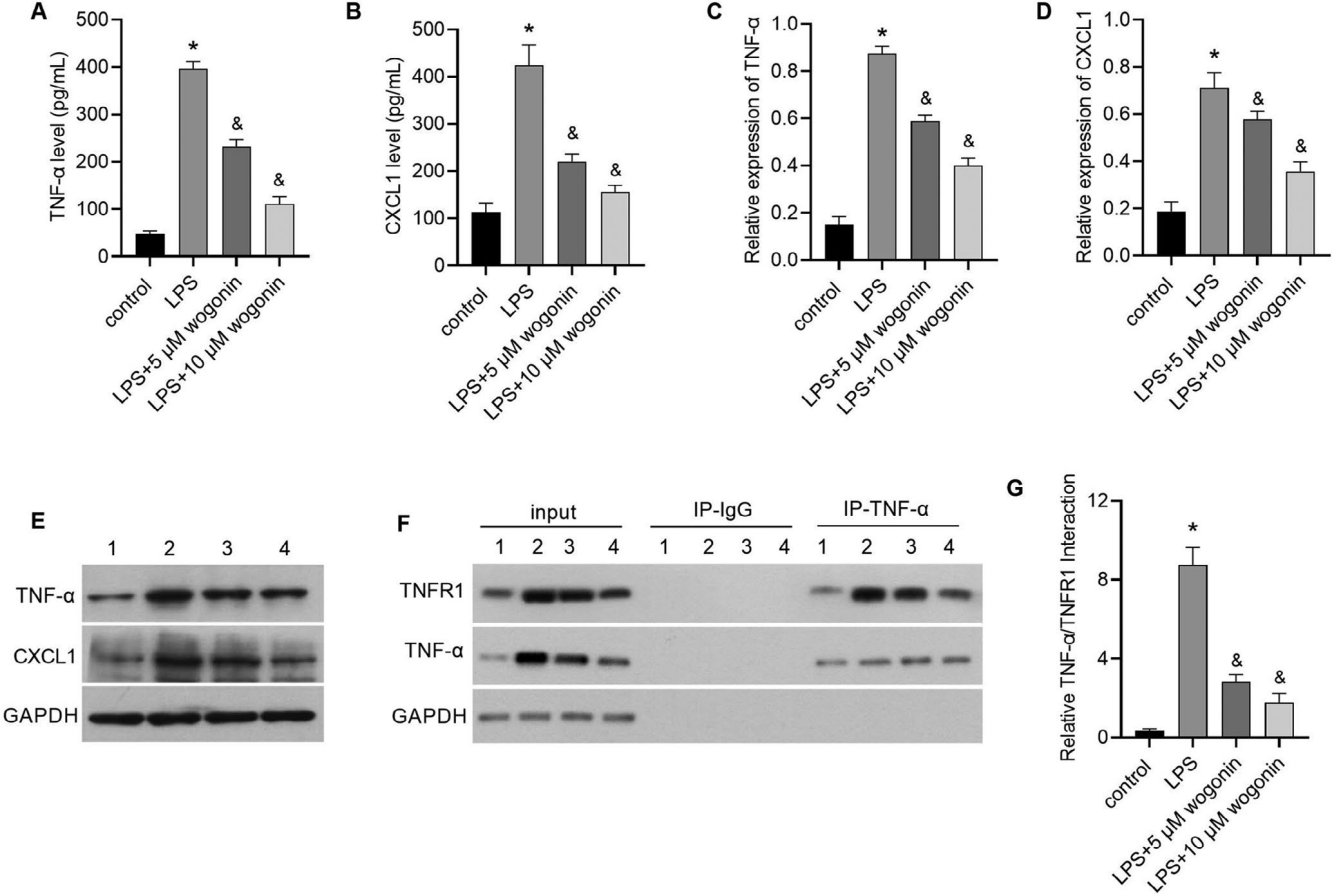
Wogonin inhibits LPS‐induced activation of TNF‐α/TNFR1/CXCL1 signalling. A‐B: Wogonin reduces LPS‐induced secretion of TNF‐α and CXCL1 in culture supernatant, as measured by ELISA. C‐E: Wogonin decreases intracellular protein expression of TNF‐α and CXCL1 in astrocytes, as detected by Western blot. Panels C and D show the statistical results of relative expression levels, while Panel E displays a representative western blot image. F‐G: Wogonin attenuates LPS‐enhanced TNF‐α–TNFR1 interaction, as analysed by co‐immunoprecipitation. Panel F shows a representative co‐IP image, and Panel G presents the statistical results of the relative levels of the TNF‐α/TNFR1 interaction. In panels E and F, numbers 1, 2, 3 and 4 represent the control group, LPS group, LPS + 5 μM wogonin group, and LPS + 10 μM wogonin group, respectively. *, *p* < 0.05 compared to the control group; &, *p* < 0.05 compared to the LPS group.

Having established that wogonin suppresses astrocyte activation, we sought to define the involved signalling pathway. We focused on the TNF‐α/TNFR1/CXCL1 axis and analysed its activation by ELISA and western blot. As shown in Figure 2A–E, LPS treatment obviously upregulated the levels of TNF‐α and CXCL1 compared to the control group. Notably, this upregulation was markedly reversed by wogonin treatment at concentrations of 5 μM and 10 μM, with a more potent effect observed at the higher concentration. Furthermore, co‐immunoprecipitation assays confirmed that LPS markedly enhanced the interaction between TNF‐α and TNFR1 (Figure 2F,G). Importantly, this enhanced interaction was attenuated in the groups co‐treated with wogonin (5 μM and 10 μM). In summary, all results demonstrated that wogonin effectively suppresses the LPS‐induced activation of TNF‐α/TNFR1/CXCL1 signalling.

### 3.3 R‐TNF‐α Blocked the Effect of Wogonin on Astrocyte Activation in Astrocytes

**FIGURE 3:**
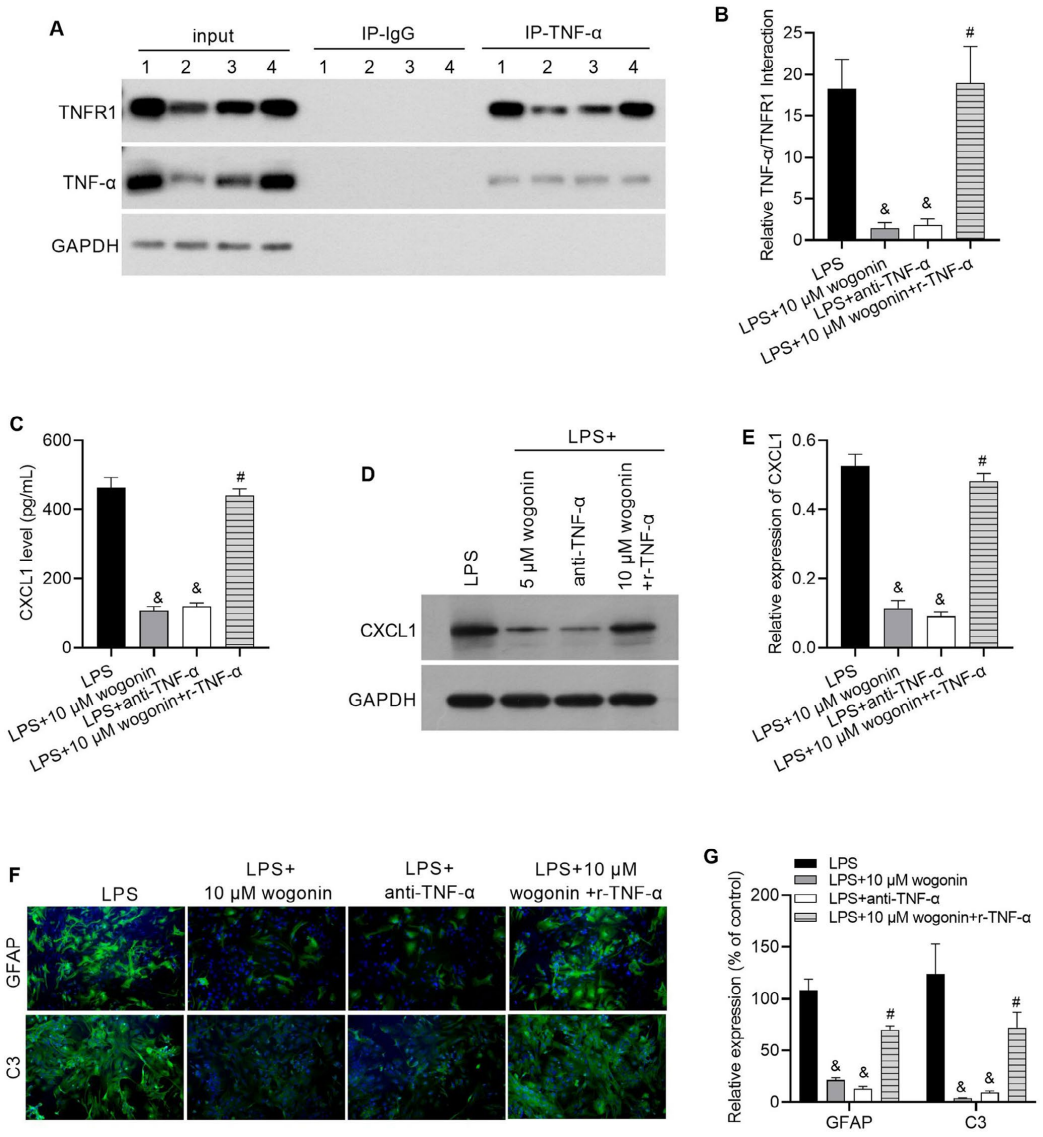
r‐TNF‐α blocks the effect of wogonin on LPS‐induced astrocyte activation. (A, B) Wogonin inhibits the interaction between TNF‐α and TNFR1, an effect that is rescued by co‐treatment with r‐TNF‐α, as assessed by co‐immunoprecipitation. Panel A shows a representative co‐IP image, and Panel B presents the statistical results of the relative levels of the TNF‐α/TNFR1 interaction. In panel A, numbers 1, 2, 3, and 4 represent the LPS group, LPS + 10 μM wogonin group, LPS + anti‐TNF‐α group, and LPS + 10 μM wogonin + r‐TNF‐α group, respectively. (C–E) Wogonin reduces CXCL1 protein levels, which is reversed by the addition of r‐TNF‐α. Panel C shows CXCL1 levels in culture medium supernatant measured by ELISA. Panel D shows the representative western blot image, while Panel E displays the statistical results of relative expression levels. (F, G) Wogonin attenuates astrocyte activation, as shown by decreased levels of GFAP and C3, and this effect is blocked by r‐TNF‐α, as detected by immunofluorescence. The left panels show representative images, and the right panels present the quantitative statistical results for GFAP and C3 expression levels. &, *p* < 0.05 compared to the LPS group; #, *p* < 0.05 compared to the LPS + wogonin group.

To investigate whether wogonin suppresses LPS‐induced astrocyte activation through the TNF‐α/TNFR1/CXCL1 pathway, rescue experiments were performed using recombinant TNF‐α (r‐TNF‐α), and the effect of anti‐TNF‐α antibody was evaluated. As shown in Figure 3A,B, both anti‐TNF‐α and wogonin treatment inhibited the interaction between TNF‐α and TNFR1. In addition, both anti‐TNF‐α and wogonin treatment reduced CXCL1 expression in LPS‐stimulated astrocytes (Figure 3C–E). Moreover, the suppressive effects of wogonin on TNF‐α–TNFR1 binding and CXCL1 expression were blocked by the co‐treatment with r‐TNF‐α (Figure 3A–E). These findings demonstrate that r‐TNF‐α rescues the inhibitory effect of wogonin on TNF‐α/TNFR1/CXCL1 signalling, and confirm the suitability of these cellular models for studying the mechanism of wogonin. Furthermore, GFAP and C3 expression levels were significantly decreased in the LPS + 10 μM wogonin and LPS + anti‐TNF‐α groups compared to the LPS group, but were elevated in the LPS + 10 μM wogonin + *r*‐TNF‐α group relative to the LPS + wogonin group (Figure 3F,G). Together, these results suggest that wogonin and anti‐TNF‐α similarly attenuate astrocyte activation, and that the anti‐activation effect of wogonin is reversed by r‐TNF‐α, indicating that wogonin may inhibit LPS‐induced astrocyte activation via the TNF‐α/TNFR1/CXCL1 signalling pathway.

### 3.4 Wogonin Alleviates Depression‐Like Behaviours in CUMS Mice, an Effect Reversed by Exogenous TNF‐α and Mimicked by TNF‐α Inhibition

**FIGURE 4:**
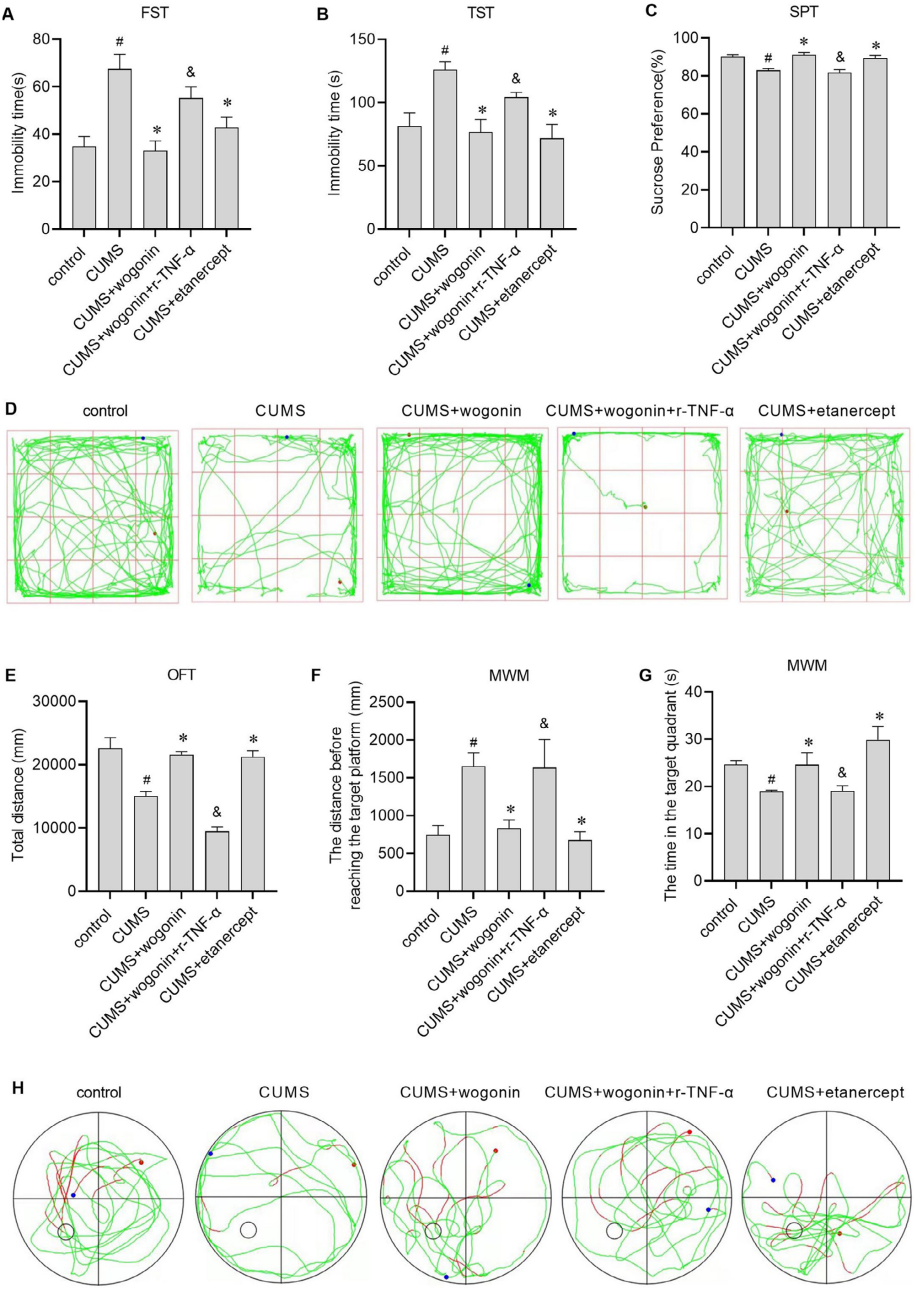
The antidepressant‐like effects of wogonin in CUMS mice are blocked by exogenous TNF‐α. (A) Immobility time in the forced swim test (FST). (B) Immobility time in the tail suspension test (TST). (C) Sucrose preference in the sucrose preference test (SPT). (D) Representative track plots in the open field test (OFT). (E) Total travel distance in the OFT. (F) The distance before reaching the target platform in the Morris water maze (MWM). (G) The time in the target quadrant in the MWM. (H) Representative swimming paths in the MWM. # indicates *p* < 0.05 compared to control group. * indicates *p* < 0.05 compared to CUMS group, & indicates *p* < 0.05 compared to CUMS+Wogonin group.

To further evaluate the antidepressant potential of wogonin in vivo, a CUMS mouse model was established, and depression‐like behaviours were assessed using a battery of behavioural tests. Compared with the CUMS group, mice treated with wogonin exhibited significantly shorter immobility times in the forced swim test (FST; Figure 4A) and tail suspension test (TST; Figure 4B), higher sucrose preference in the sucrose preference test (SPT; Figure 4C), and increased total distance travelled in the open field test (OFT; Figure 4D,E). In the Morris water maze (MWM; Figure 4F–H), mice treated with wogonin also showed a reduced distance before reaching the target platform and prolonged time spent in the target quadrant. These results demonstrate that wogonin treatment markedly alleviates depression‐like behaviours in CUMS‐induced depressive mice. Furthermore, the suppressive effect of wogonin on depression‐like behaviours was reversed by co‐administration of recombinant TNF‐α (r‐TNF‐α), and the TNF‐α inhibitor etanercept produced effects comparable to those of wogonin (Figure 4). Together, these findings suggest that the TNF‐α pathway may be critically involved in the behavioural effects of wogonin.

### 3.5 Wogonin Rescues CUMS‐Induced Synaptic Plasticity Impairments, an Effect Blocked by Exogenous TNF‐α and Mimicked by TNF‐α Inhibition

**FIGURE 5:**
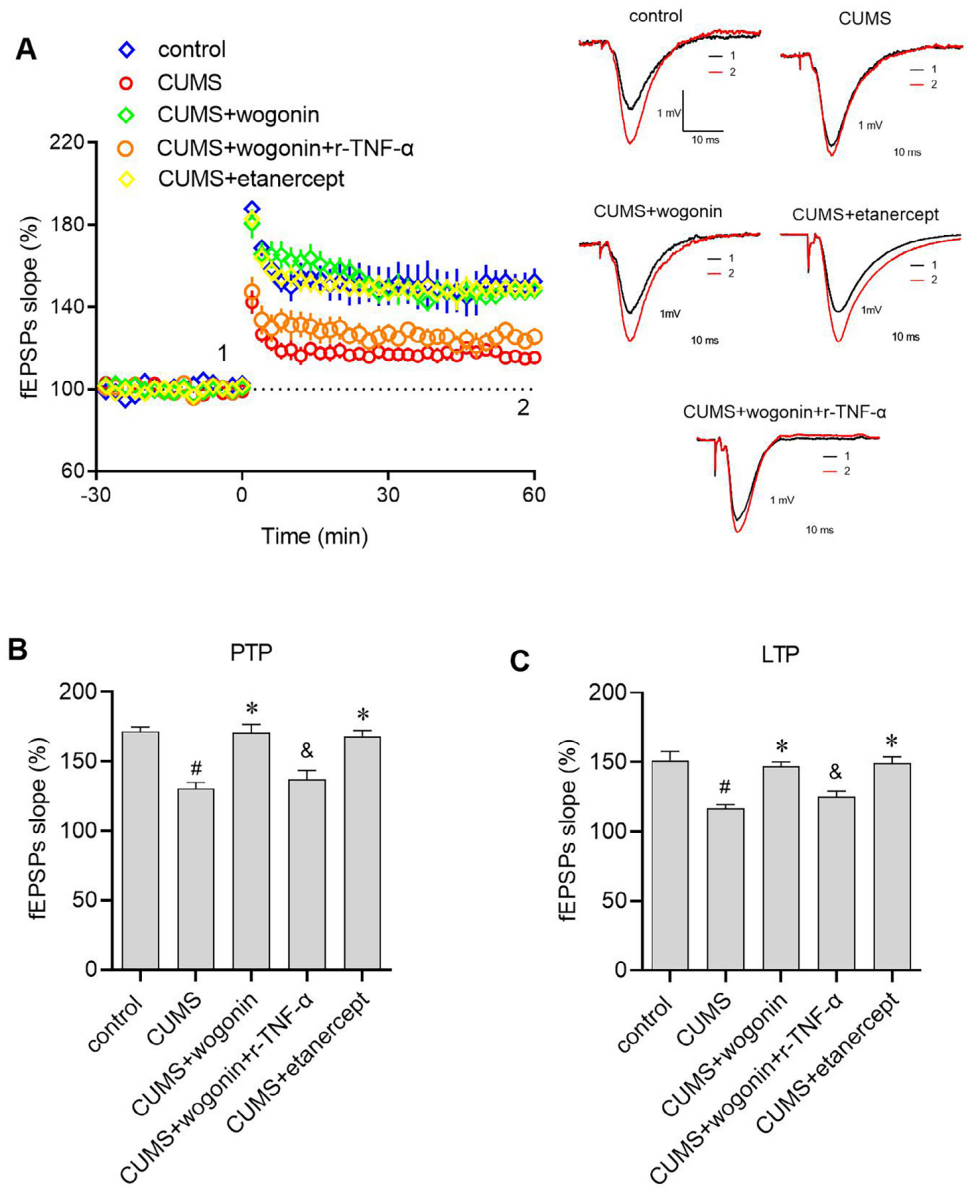
The restorative effect of wogonin on CUMS‐impaired synaptic plasticity is reversed by exogenous TNF‐α. (A) Time course of normalized fEPSP slopes recorded in the hippocampal CA1 region before (−30 to 0 min; baseline) and after (0 to 60 min) theta‐burst stimulation (TBS). Traces on the right show representative fEPSPs before (black) and after (red) TBS. (B, C) Quantitative analysis of PTP (1–5 min post‐TBS) and LTP (50–60 min post‐TBS). #*p* < 0.05 versus control group; *p < 0.05 versus CUMS group; &*p* < 0.05 versus CUMS + wogonin group.

To investigate whether wogonin modulates synaptic plasticity in the CUMS mouse model of depression and to determine if its effects involve the TNF‐α pathway, we assessed hippocampal synaptic function by measuring field excitatory postsynaptic potentials (fEPSPs), post‐tetanic potentiation (PTP) and long‐term potentiation (LTP), key electrophysiological measures used to quantify and understand synaptic transmission and plasticity [27]. Our results demonstrated that CUMS exposure significantly reduced fEPSP slope (Figure 5A), PTP (Figure 5B) and LTP (Figure 5C) compared to the control group, while wogonin treatment effectively restored these synaptic plasticity parameters. Notably, the effects of wogonin were reversed by co‐administration of r‐TNF‐α, and the TNF‐α inhibitor etanercept produced improvements comparable to wogonin. These data indicate that modulation of TNF‐α signalling is tightly associated with the beneficial effects of wogonin on hippocampal synaptic plasticity.

### 3.6 Wogonin Mitigates CUMS‐Induced Neuronal Damage and Astrocyte Activation, Effects Counteracted by Exogenous TNF‐α and Mimicked by TNF‐α Inhibition

**FIGURE 6:**
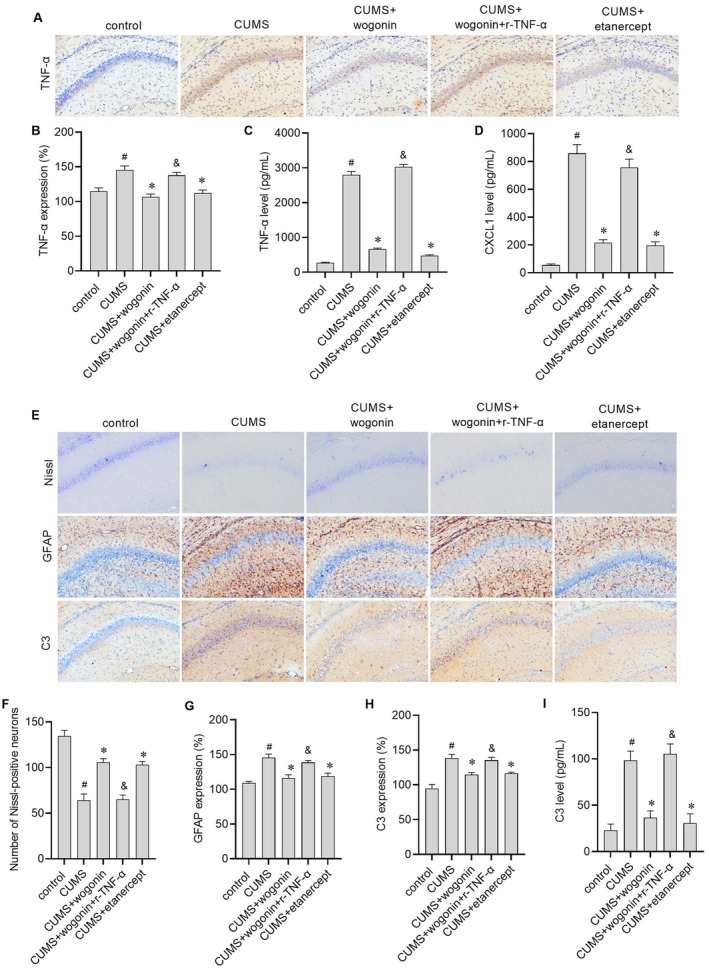
The neuroprotective and astrocyte activation‐inhibiting effects of wogonin in CUMS mice are reversed by exogenous TNF‐α. (A) Representative immunohistochemical images of TNF‐α in the hippocampal CA1 region. (B) Quantitative analysis of TNF‐α expression in the hippocampus. (C, D) TNF‐α and CXCL1 levels in cerebrospinal fluid measured by ELISA. (E) Representative images of Nissl staining showing neuronal morphology and Nissl body density and immunohistochemical images of GFAP and C3in the hippocampal CA1 region. (F) Statistic analysis of Nissl‐positive neuron number. (G, H) Quantitative analysis of GFAP and C3 expression in the hippocampus via immunohistochemistry. (I) C3 level in cerebrospinal fluid measured by ELISA. #*p* < 0.05 versus control group; *p < 0.05 versus CUMS group; &*p* < 0.05 versus CUMS + wogonin group.

To further investigate whether wogonin exerts its effects on depression‐like behaviour in CUMS mice through the TNF‐α/TNFR1/CXCL1 signalling pathway involved in astrocyte activation, we measured the levels of TNF‐α and CXCL1. Compared to the CUMS group, the TNF‐α level in the hippocampal CA1 region (Figure 6A,B) and both TNF‐α and CXCL1 levels (Figure 6C,D) in the cerebrospinal fluid were significantly reduced after wogonin treatment. These results showed that wogonin can also inhibit TNF‐α/TNFR1/CXCL1 signalling in vivo. The results of Nissl staining showed that wogonin treatment alleviated CUMS‐induced neuronal injury (Figure 6E). Nissl bodies were clearly visible and abundant in the control group, whereas their numbers were noticeably reduced in the CUMS group (Figure 6E,F). In contrast to the CUMS group, the number of Nissl bodies was significantly increased in the CUMS + wogonin group (Figure 6E,F). In addition, GFAP and C3 expression levels were decreased after wogonin treatment, suggesting that wogonin mitigates the astrocyte activation induced by CUMS (Figure 6E,G–I). Critically, the neuroprotective and anti‐astrocytic activation effects of wogonin were reversed by co‐administration of r‐TNF‐α, while the TNF‐α inhibitor etanercept alone produced effects similar to wogonin. These results position TNF‐α as a key upstream modulator in the CUMS‐induced pathological cascade that can be targeted by wogonin.

## 4 Discussion

Astrocytes are strongly implicated in the pathophysiology of depression, where their dysfunction can disrupt synaptic formation and plasticity [28, 29]. Therefore, we first analysed the effect of wogonin on LPS‐induced astrocyte activation by measuring the expression of C3 and GFAP. GFAP is an intermediate filament protein that serves as a classical marker for astrocyte activation [30]. Astrocyte activation is heterogeneous, and activated astrocytes are traditionally classified into two types: neurotoxic A1 reactive astrocytes and neuroprotective A2 reactive astrocytes [31, 32]. C3 plays a crucial role in the immune system by participating in the complement cascade [33], its expression is specifically elevated in A1 astrocytes. Thus, C3 is regarded as one of the most specific markers for identifying the neurotoxic A1 reactive astrocyte phenotype [32]. Our results demonstrated that wogonin effectively suppresses astrocyte activation, as supported by its obvious inhibition of both GFAP and C3 expression in LPS‐induced astrocytes and the hippocampus of CUMS mice.

Interestingly, the inhibitory effect of wogonin on LPS‐induced C3 expression in astrocytes was not linear, with maximal inhibition observed at 5–10 μM and a slight reduction in efficacy at 20 μM. This biphasic effect is not uncommon for natural compounds, including wogonin [26, 34]. Gene expression is not isolated but is regulated through complex, interconnected networks. Natural compounds can simultaneously influence the expression of multiple genes, which in turn interact with each other within intricate regulatory circuits. We speculate that different concentrations of wogonin may engage distinct regulatory networks in astrocytes. These networks could directly or indirectly influence C3 expression, thereby accounting for the observed non‐linear dose–response. Further investigation into the precise mechanisms by which wogonin regulates C3 expression will be crucial for elucidating this phenomenon.

Importantly, we systematically investigated the effects of wogonin on depression‐like behaviour in CUMS mice by performing the SPT, TST, FST, MWM and OFT. CUMS mice treated with wogonin exhibited higher sucrose preference in the SPT and shorter immobility time in the TST and FST. The SPT is an animal behavioural experiment used to investigate depressive behaviour. Rodents have a natural preference for sweetness; however, when mice exhibit depression‐like behaviour, their preference for sugar‐water is reduced. The TST and FST are two commonly used methods to evaluate antidepressant effects, and both are important behavioural paradigms for evaluating the behavioural despair phenotype after depression modelling. As such, the results of the SPT, TST and FST revealed that wogonin treatment increased happiness and reduced despair in CUMS mice. The OFT assesses spontaneous behaviour, exploratory activity and anxiety levels in experimental animals exposed to a new environment, while the MWM test assesses spatial learning and memory. We found that CUMS mice treated with wogonin spent more time in the target quadrant and travelled shorter distances before entering the target quadrant in the MWM. Further, in the OFT, CUMS mice treated with wogonin travelled shorter distances. These results suggested that wogonin treatment alleviated CUMS‐induced impairments in spatial learning and memory, thus demonstrating that wogonin mitigates depression‐like behaviour in CUMS‐induced mouse models.

Moreover, wogonin treatment prevented the CUMS‐induced suppression of synaptic plasticity in the hippocampal CA1 region, as evidenced by the rescue of the fEPSP slope, PTP and LTP. fEPSP, PTP and LTP are important manifestations of synaptic plasticity [27]. fEPSPs reflect the overall strength of synaptic input to a population of neurons. LTP is a long‐lasting enhancement of synaptic transmission, typically lasting hours to years, involving persistent changes in both presynaptic and postsynaptic cells, and is generally considered the neural basis for learning and memory. Conversely, PTP is defined as the short‐term enhancement of synaptic transmission, usually lasting from seconds to minutes, occurring following strong stimulation. Collectively, these findings demonstrate that wogonin can reduce CUMS‐induced impairments in neuroplasticity, and that this may be the neural basis underlying the wogonin‐mediated mitigation of depression‐like behaviour in CUMS‐induced mouse models.

To test the hypothesis that wogonin exerts its effects via the TNF‐α/TNFR1/CXCL1 axis, we investigated this pathway in our models. Our data show that both LPS treatment and CUMS exposure activate this pathway, and that wogonin treatment effectively reverses this activation. Crucially, the beneficial effects of wogonin were abolished by co‐administration of recombinant TNF‐α (r‐TNF‐α). These results collectively suggest that wogonin mitigates depression‐like behaviors in CUMS mice primarily by inactivating the TNF‐α/TNFR1/CXCL1 signalling axis in astrocytes.

It is well recognized that the prefrontal cortex (PFC) plays a central role in emotion and cognition in depression [35]. In this study, we focused on the hippocampal CA1 region because it also plays a key role in depression—it shows volume loss in patients and impaired synaptic plasticity (LTP) in animal models [35]. The CA1 region also provides a reliable model for LTP recording in brain slices, which helps us study mechanisms precisely. We also analysed cerebrospinal fluid (CSF), which reflects whole‐brain inflammation and is anatomically linked to the hippocampus [36, 37], providing a complementary systemic perspective to support our local CA1 findings. While focusing on CA1 is a limitation, future work will extend to other regions such as the PFC.

## 5 Conclusions

Wogonin mitigates depression, and wogonin may play its role by inhibiting TNF‐α/TNFR1/CXCL1 signalling‐mediated astrocyte activation. Overall, our study suggests that wogonin may be an effective treatment option for depression.

## Author Contributions

H.C.: Writing – original draft, methodology, formal analysis, conceptualization. K.Z.: Writing – original draft, methodology, formal analysis, conceptualization. W.H.: Conceptualization, writing – review and editing, funding acquisition, project administration. B.L.: Conceptualization, writing – review and editing, funding acquisition, project administration. All authors have read and agreed to the published version of the manuscript.

## Funding

This work was supported by Guangdong Provincial Medical Science and Technology Research Fund, B2024056. Scientific Research and Development Fund of Dongguan People's Hospital, K202023.

## Ethics Statement

All animal experimental procedures were approved by the Experimental Animal Ethics Committee of Guangzhou Laian Technology Co. LTD.

## Conflicts of Interest

The authors declare no conflicts of interest.

## Data Availability

The data that support the findings of this study are available from the corresponding author upon reasonable request.
